# Genetic Diversity and Population Structure of *Juniperus seravschanica* Kom. Collected in Central Asia †

**DOI:** 10.3390/plants12162961

**Published:** 2023-08-16

**Authors:** Moldir Yermagambetova, Shyryn Almerekova, Orzimat Turginov, Ormon Sultangaziev, Saule Abugalieva, Yerlan Turuspekov

**Affiliations:** 1 Institute of Plant Biology and Biotechnology, Almaty 050040, Kazakhstan; m.ermagambetova@ipbb.kz (M.Y.); s.almerekova@ipbb.kz (S.A.); s.abugalieva@ipbb.kz (S.A.); 2 Faculty of Biology and Biotechnology, Al-Farabi Kazakh National University, Almaty 050040, Kazakhstan; 3 Institute of Botany, Tashkent 100125, Uzbekistan; orzimat@mail.ru; 4 Fauna and Flora International, Bishkek 720005, Kyrgyzstan; ormon@list.ru

**Keywords:** *Juniperus seravschanica*, genetic diversity, population structure, simple sequence repeat (SSR), Central Asia

## Abstract

*Juniperus seravschanica* Kom. is a species that grows widely in the mountain ranges from Central Asia to Oman. It is an important tree for the formation of shrub–forest massifs in mountainous areas and for draining and fixing soils from middle to high altitudes. A comprehensive study of the species’ genetic diversity and population structure is a basic approach to understanding the current status of *J. seravschanica* resources for the development of future conservation strategies. Samples from 15 populations of *J. seravschanica* were collected from the mountain ranges of Uzbekistan, Kyrgyzstan, and Kazakhstan. The genetic diversity and population structure of 15 Central Asian populations of *J. seravschanica* were assessed using 11 polymorphic simple sequence repeat (SSR) markers. Genetic diversity parameters, including the number of alleles (na), the effective number of alleles (ne), Shannon’s information index (I), the percentage of polymorphic loci (PPL), Nei’s genetic diversity index (Nei), principal coordinate analysis (PCoA), etc., were evaluated. The analysis of 15 *J. seravschanica* populations based on 11 polymorphic SSRs detected 35 alleles. The average PIC value was 0.432, and the highest value (0.662) was found in the JT_40 marker. Nei’s genetic diversity index for the *J. seravschanica* populations was 0.450, ranging from 0.407 (population 14) to 0.566 (population 4). The analysis of molecular variance (AMOVA) showed that 90.3% of total genetic variation is distributed within the population. Using the alleles of all the populations, the gene flow (Nm) was found to be 4.654. Population structure analysis revealed poor clustering in the studied populations and confirmed our AMOVA results. The output of this work can be efficiently used for the maintenance of the species across the Central Asian region.

## 1. Introduction

Species of the genus *Juniperus* L., belonging to the Cupressaceae family, are common in the mountain ranges of Central Asian countries, including Kazakhstan (seven species), Kyrgyzstan (4), Tajikistan (4), and Uzbekistan (3) [1]. They grow as shrubs or trees and play an essential ecological role in forming shrub–forest massifs in mountainous areas and draining and fixing soils on mountain ranges. One common *Juniperus* species in this region is *Juniperus seravschanica* Kom. *J. seravschanica* is a tall, wind-pollinated, drought-resistant coniferous evergreen tree that is important as a medicinal plant. It is a source of cones, raw materials for producing essential oils (16.8% cedrol content), and leaves that are widely used in medicine [2]. The name of this species is associated with the region along the Zeravshan (Zarafshon) River in Uzbekistan and Tajikistan, where this tree was initially found, widely spread across the hills of the Pamir-Alay mountain system [3]. Therefore, it was assumed that *J. seravschanica* originated in that area and spread to the neighboring countries of Kyrgyzstan, Kazakhstan, Turkmenistan, Afghanistan, Pakistan, India, Iran, and Oman [4]. This species is the main forest-forming factor of the Western Tian Shan (Kazakhstan, Kyrgyzstan) and the Pamir-Alay (Kyrgyzstan, Tajikistan, Uzbekistan) mountain systems [5]. Juniper forests with *J. seravschanica* as the dominant species are typically found on dry foothills or at middle to high altitudes. This species is a key woodland component from 1000 to 3500 m above sea level [5]. *J. seravschanica* is often found in steep and sagebrush slopes, most often those with southern and western exposures, and frequently in rubbly–stony mountain gray-brown (xeromorphic) soils underlain by dense rocks. This species can grow on a wide variety of substrates, from steep (up to 70°) rocky outcrops to nutrient-rich brown soils with a significant (up to 16%) humus content. This property plays an essential ecological role in water and soil protection, preventing destructive mudflows on mountain ranges [6]. Despite the wide distribution of this species in the region, a decrease in the number of natural populations has been observed. The main factors in the species’ decline are environmental changes, the use of wood for construction, overgrazing, and fires [7]. Therefore, in order to preserve its natural population, this species was listed in the Red Book of Central Asia [8], including in Kazakhstan [9,10]. Therefore, the conservation of this species’ genetic resources and the reconstruction of its habitats are essential. A comprehensive assessment of the genetic diversity and genetic structure of this species’ natural populations will serve as a basic background for proposing conservation strategies and protecting *J. servaschanica* resources [4,11,12].

Taxonomically, *J. seravschanica* belongs to the *Juniperus* section *Sabina*, which was first described by Komarov in 1932 [13]. A distinctive feature of *J. seravschanica* is globular, blackish-blue fleshy 9–12 mm long fruits or cone-berries. The species *J. excelsa*, *J. polycarpos* K. Koch, and *J. seravschanica* Kom. have difficulties in identification due to their morphological characteristics. According to Adams et al. [14] *J. excelsa* is distributed from Greece to Turkey, *J. polycarpos* grows in Azerbaijan and Lebanon, and the distribution area of *J. seravschanica* stretches from Central Asia to Oman. Due to existing taxonomic uncertainties, it is important to study these species using polymorphic molecular markers [15]. Within the genus, a sufficiently large number of reports were dedicated to the evaluation of the genetic diversity of different *Juniperus* species [16,17,18,19,20,21,22,23,24,25,26]. Similarly, *J. seravschanica* was extensively studied for the determination of the area for core and peripheral populations [27], morphometric traits [13], chemical components [28], and essential oils [2,15]. Additionally, the complete chloroplast genome sequence of this species has been reported [29]. Nevertheless, there is a shortage of information on the evaluation of the genetic diversity and structure of the populations distributed in the Central Asian region.

Recent studies show that genetic diversity patterns significantly affect the viability and resistance of environmental conditions of local populations [30,31]. The importance of stochastic factors (gene flow, genetic drift, and founder events) and environmentally specific natural selection may change from a species’ core toward its boundary. Increased genetic drift caused by small population size and isolation at the species periphery leads to a loss of genetic variation, but may also promote population differentiation [32]. Thus, identifying patterns of intraspecific diversity within regions is vital for our understanding of ecological and evolutionary (e.g., speciation) processes [33], as well as for setting conservation priorities [34]. In addition, a comparative assessment of the genetic diversities of core and peripheral populations is relevant, as the original populations of a species often differ in the ecological conditions in which they live. Historically, the genetic diversity of the *Juniperus* genus populations was actively studied using different informative DNA markers, including RAPDs (randomly amplified polymorphisms of DNA) [35,36], AFLPs (amplified fragment length polymorphisms) [37], SSRs (simple sequence repeats) [21,26], etc. In particular, SSR markers, or microsatellites, can be very informative due to their wide distribution within genomes, high degree of polymorphism, good duplicability, and codominant inheritance [26,38,39,40,41,42]. However, the genetic diversity of *J. seravschanica* has only been assessed using a few types of DNA markers. For example, Adams [15], Sultangaziev, et al. [5], and Rahimian Boogar and Salehi [43] studied selected populations of the species using RAPD, PCR-RFLP, and ISSR markers, respectively. Naturally, assessing these populations using different types of DNA markers cannot provide a comprehensive platform for evaluating genetic diversity in Central Asia. Therefore, this study aimed to analyze the genetic diversity of *J. seravschanica* populations from mountain ranges in three Central Asian countries (Kazakhstan, Kyrgyzstan, and Uzbekistan) using polymorphic SSR markers. It was assumed that this investigation might help evaluate the variability of SSR markers in this species, and identify genetically diverse populations for the development of future strategies associated with the maintenance of *J. seravschanica* resources in Central Asia.

## 2. Results

### 2.1. Polymorphism of Tested SSR Markers

The initial sample screening with a set of 18 SSR markers demonstrated that 11 out of the 18 tested SSR markers were polymorphic and suitable for genetic analysis (Table 1).

Genotyping of a total of 323 samples from 15 populations with 11 SSRs (Table S1) resulted in the identification of 35 alleles. The number of alleles per SSR marker ranged from 2 to 5, with an average of 3.2 alleles per locus. JT_37 and JT_40 markers amplified the ultimate number of alleles (5 alleles), while Jce05, JT_33, and JT_34 generated the lowest number of alleles (2 alleles) (Table 1). The average PIC value was 0.432, ranging from 0.077 (Jce05) to 0.662 (JT_40), and the lowest and highest level of heterozygosity 0.081 and 0.712 at loci Jce_05 and JT_40, respectively (Table 1).

### 2.2. Genetic Diversity in Collected Populations of J. seravschanica in Three Central Asian Countries

The average number of alleles amplified using 11 polymorphic SSR loci in the 15 studied populations was 2.6 and ranged from 2.2 (population 2) to 3 (population 12) (Table 2). The number of effective alleles (ne) ranged from 1.7 to 2.3, with an average value of 1.9. The average PPL was 91.5% and varied from 82% to 100%. The average number of observed heterozygosity (Ho) and expected heterozygosity (He) were 0.695, and 0.575, respectively. The mean Nei’s genetic diversity index for studied *J. seravschanica* populations was 0.450, ranging from 0.407 (population 14) to 0.566 (population 4) (Table 2). The highest average Nei’s index value was recorded in the populations collected in Uzbekistan (0.473), followed by those from South Kyrgyzstan and Kazakhstan (both 0.443). Interestingly, the assessment of the *ne* and PPL indices shows that the highest values were recorded for the populations from Kazakhstan (Table 2).

The results of the AMOVA suggest that 90.3% of total genetic variation is distributed within, and 9.7% between population groups, respectively. The gene flow (Nm) was calculated using the F_st_ values and equals 4.654 migrants per generation (Table 3).

**Figure 1:**
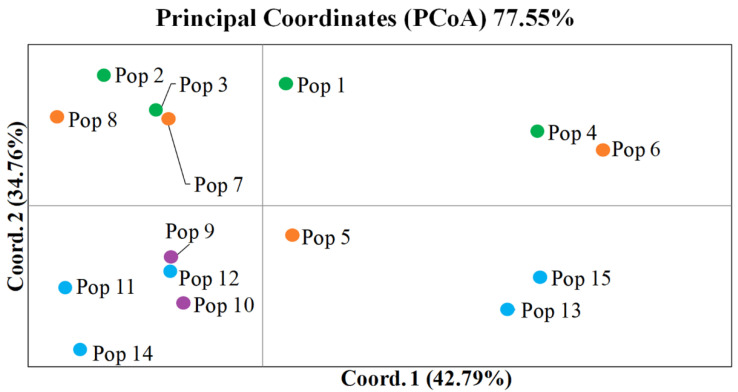
Principal coordinates analysis (PCoA) of 15 populations of *Juniperus seravschanica* from Central Asia based on pairwise population differentiation (PhiPT) values. Notes: Circles highlighted as green—Uzbekistan, orange—South Kyrgyzstan, purple—North Kyrgyzstan, blue—Kazakhstan.

The PCoA plot shows the distances of the *J. seravschanica* populations using two principal coordinates: Coordinate 1 and Coordinate 2, which explain 42.79% and 34.76% of the total variation among populations, respectively (Figure 1). Coordinate 1 effectively separates populations 4, 6, 13, and 15 from the majority of the other populations. Coordinate 2 separates the populations of Uzbekistan and South Kyrgyzstan from the populations of North Kyrgyzstan and Kazakhstan, except for population 5, which was grouped together with populations from North Kyrgyzstan and Kazakhstan (Figure 1). The closest population to the intersection of eigenvalues 1 and 2 was population 5 from South Kyrgyzstan (Figure 1). The Mantel test for correlation between population genetic distance (PopGD) and geographic distance (GGD) matrices (r^2^) was equal to 0.0885 (*p* > 0.020). The correlation between Nei’s genetic distance (NeiP) and the geographic distance (GGD) was 0.0082 (*p* > 0.270).

The results of SSR analysis for the fifteen *J. seravschanica* populations were also evaluated using an unrooted dendrogram based on the unweighted pair-group method and the arithmetic means (UPGMA) method (Figure S1). The dendrogram separates the studied populations into three groups: I, II, and III (Figure S1). Groups I and II form the first clade, and populations in group III are separated into the second clade. The clustering results are concordant with the PCoA plot (Figure 1).

### 2.3. The Genetic Structure of J. seravschanica Based on the Analysis of Fifteen Populations from Central Asia

The analysis of the STRUCTURE output suggests poor clustering for the 15 studied populations collected from four different regions (Figure S2), which confirmed the AMOVA results for partitioning total genetic variation within and between populations (Table 3). The Structure Harvester analysis using the “elbow” method indicates that the most significant K steps were K3 and K5 (Figure S2). At the K3 step, cluster 3 mostly consisted of plants from three populations from Uzbekistan, one population from South Kyrgyzstan, two populations from North Kyrgyzstan, and three populations from Kazakhstan. At this K step, cluster 1 was mostly populated by plants from three populations from South Kyrgyzstan and two populations from Kazakhstan. Notably, population 4, from Uzbekistan, had an equal number of plants in both clusters (Table 4). A minor number of plants in all four regions represented cluster 2. At the K5 step, the plant ratio between clusters was slightly modified, as most plants were part of clusters 4 and 5 (Table 4). Cluster 3 was heavily represented by plants from population 4 (41.7%). The majority of plants in population 1 from Uzbekistan (45.5%) and population 7 from South Kyrgyzstan (52.4%) were part of cluster 2. Finally, cluster 1 was strongly represented by plants in population 5 from South Kyrgyzstan (30.8%). Notably, the plants in population 5 were equally represented in clusters 1, 4, and 5 (Table 4), which is in good agreement with the centralized position of this population in the PCoA plot (Figure 1).

## 3. Discussion

The genetic diversity and population structure of forests are shaped by past historical and current human-mediated processes. While its assessment is crucial to inform conservation and forestry management, the geographic scale at which it is quantified plays an essential role in developing species conservation strategies. We carried out a genetic diversity analysis of 15 populations of *J. seravschanica* originating from three Central Asian countries (Kazakhstan, Kyrgyzstan, and Uzbekistan) using 11 polymorphic SSR markers (Table 2). The estimated average number of alleles (3.2) identified in this study was comparable to those of *J. cedrus* reported by Rumeu et al. [20] and *J. thurifera* reported by Teixeira et al. [21], but lower than the *J. sabina* (6.70) reported by Lu et al. [26], *J. excelsa* (12.57) reported by Evren and Kaya [25] or *J. communis* (9–23 alleles) species reported by Michalczyk et al. [17]. The average Shannon’s diversity index (I) calculated using SSR markers was 0.703, higher than the index (0.428) calculated using RAPD markers [44]. Accordingly, 91.5% was the PPL percentage value determined utilizing SSR markers.

The mean genetic diversity for this species (0.450), including for peripheral populations in Kazakhstan (0.443), was relatively high (Table 2). A species’ resistance to environmental change is stronger the more genetic diversity it has [26,45]. The total genetic variation of *J. seravschanica* populations collected from mountain ranges of three Central Asian countries was divided into 90.3% within populations and 9.7% between populations (Table 3).

The values of Nm in conifers have a tendency to be much higher, with values of Nm ≥ 3 being the norm [46]. Our results indicate a high level of gene flow for this species-Nm-4.654), which is higher than the mean values of gene flow for other congeneric juniper species—*J. communis*—1.09 [47] and *J. brevifolia*—2.43 [48], suggesting a high level of historical gene flow between these populations. We believe that gene flow via wind-dispersed pollen is less likely to reach larger distances, as reported for *J. tibetica* by Opgenoorth [49], and does not exceed 2 km. Moreover, often isolated mountain slopes with deep valleys restrict pollen movement outside of that territory. Furthermore, frequent rains during the pollination period wash away the pollen; therefore, low levels of fertile seeds have been reported [50]. It is more likely that gene flow happens mainly via fleshy female cones, preferred mainly by birds from *Turdus* and *Fringillidae,* who feed on cones during the breeding season and disseminate seeds for a longer distance [51]. Similar patterns have been reported for other juniper species [52]. According to Slatkin [53], Nm of more than four migrants/ generation prevents genetic differentiation between populations, a pattern we observe for *J. seravschanica* in the Central Asian regions.

Our assessment of the STRUCTURE outputs (Table 4 and Table S2) for different K steps shows a very poor structuration of the populations in this species, which is in good agreement with our AMOVA results (Table 3). Nevertheless, the STRUCTURE plot during the K3 step suggests that the collected populations were separated into two groups. The first group consisted of three populations from Uzbekistan (populations 1–3), one from South Kyrgyzstan (population 8), two from North Kyrgyzstan (populations 9 and 10), and two from Kazakhstan (populations 12 and 14). The second group consisted of one population from Uzbekistan (population 4), three from South Kyrgyzstan (populations 5–7), and two from Kazakhstan (populations 14 and 15) (Table 4). This separation possibly indicates gene flow routes from the southern to northern territories of the region, as the highly populated Ferghana valley was found to be a significant barrier for gene flow between northern and southern sub-populations in Kyrgyzstan [5]. The gene flow is likely happening through the territory of Uzbekistan, more precisely via the Gissar and Western-Tianshan mountain ranges, where the highest average level of genetic diversity was registered (Table 2). Nevertheless, analysis of the plot from the K5 step suggests an outstanding role of population 5 in South Kyrgyzstan (Table 4), where plants were evenly distributed with high frequency in three out of five clusters. Additionally, the eigenvalues for population 5 are in the middle of those for Coordinates 1 and 2 in the PCoA plot (Figure 1). This result is unsurprising, as this population was collected in the Turkestan Range, just north of the Zeravshan River.

A high gene flow among populations probably influenced the relatively high level of genetic diversity in the populations from Kazakhstan. Although these populations grow on the edge of the area of *J. seravschanica* distribution, it was determined that their level of genetic diversity is equal to the values of populations from South Kyrgyzstan (Table 2). There are three main hypotheses in the literature regarding trends in genetic diversity along core–peripheral clines, each with different spatial implications [54]. The first hypothesis suggests that diversity increases from the periphery to the core, the “Carson hypothesis”; the second one suggests that diversity decreases from the periphery to the core, the “Fischer hypothesis”; and the third one suggests homogeneous diversity from the periphery to the core, the “Mayr hypothesis”. The results of this study support the third hypothesis, which, in this particular case, was based on (1) species distribution limited to the mountain chains of three neighboring countries in Central Asia, and (2) a high level of gene flow. A review of the literature indicates that the hypothetical region for the core populations of *J. seravschanica* is the mountain ranges of northern Pamir-Alay (Zaamin National Park, Zeravshan River) in Uzbekistan [12,55], although none of the populations from Tajikistan have been assessed so far. The results of this study suggest that the region for the core population of this species might be wider and may include the Turkestan Range in South Kyrgyzstan. The regions for the northern peripheral populations in Kazakhstan and North Kyrgyzstan collected in this work are possibly a natural northern border for the species distribution. Further north and northeast, no *J. seravschanica* populations were found, which indicates that Kazakhstan’s colder climate is unsuitable for this species’ growth. Thus, the population structure outputs and genetic diversity data in this work revealed a relatively high rate of gene flow [46,47,48] in the species and a high level of genetic diversity within the populations (90.3%). The results of this study can be used to develop species conservation strategies, as well as afforestation programs across the Central Asian region.

## 4. Materials and Methods

### 4.1. Sampling Area and Approaches

**Figure 2:**
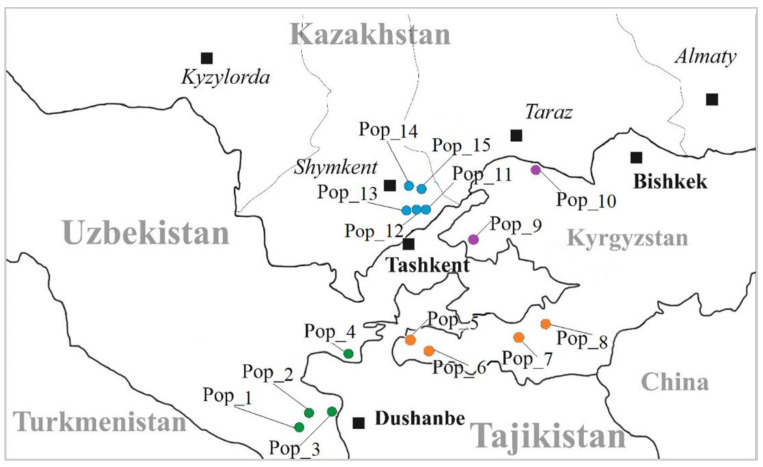
Location of sampled *Juniperus seravschanica* populations in Central Asia. Pop—population; enumeration according to Table 1. Notes: Circles highlighted as green—Uzbekistan, orange—South Kyrgyzstan, purple—North Kyrgyzstan, blue—Kazakhstan.

Across the three Central Asian countries (Kazakhstan, Kyrgyzstan, and Uzbekistan) 323 individuals of 15 *J. seravschanica* populations were sampled at altitudes from 1420 to 2293 m above sea level (Figure 2; Table 5). The number of samples in each population ranged from 10 (Population 1) to 43 (Population 15). The number of samples per population varied significantly due to difficulties related to the inaccessibility of terrains and population sizes.

The minimum distance of collected plant materials in the population ranged from 30 to 50 m between individuals, each individual was labeled and dried in silica gel and stored at room temperature until DNA was extracted. The geographical coordinates of each population are listed in Table 5. Due to the distinct geographic spread of *J. seravschanica* in Kyrgyzstan, the populations in the country were separated into two groups—North and South Kyrgyzstan (Figure 2, Table 5).

### 4.2. DNA Extraction and SSR Analysis

For each sample, total genomic DNA was extracted from 50 to 60 mg of dried leaves, which were ground to powder using two steel beads and a shaking mill (Retsch MM301, Haan, Germany), following the cetyltrimethylammonium bromide (CTAB) method [56]. The quality of the extracted DNA was checked using 1% Tris-borate-EDTA (TBE) agarose gels and NanoDrop 2000 spectrophotometer (Thermo Fisher Scientific, Waltham, MA, USA). The concentration of the DNA samples was normalized at 100 ng/μL and stored at –20 °C for further SSR analysis.

Eighteen primer pairs designed for the amplification of SSR loci for other *Juniperus* species, namely *J. cedrus* [20], *J. thurifera* [21], and *J. sabina* [26] were chosen to test their transferability for congeneric species—*J. seravschanica*. Information on the primer sequences, repeat types, and amplification conditions of the juniper microsatellites is provided in Table 6.

The DNA was amplified in a thermal cycler (Thermo Fisher Scientific, Waltham, MA, USA) as follows: denaturation at 95 °C for 3 min; 35 cycles consisting of 30 s of denaturation at 95 °C; a 30 s annealing at temperatures ranging from 54 to 63 °C depending on the primer pair used; and a 90 s extension at 72 °C, followed by a final extension at 72 °C over 20 min. The PCR reactions were performed in a volume of 20 μL containing 1 × PCR buffer, 1.5 mM of MgCl_2_, 0.5 mM each of dNTPs, 0.2 μM of each primer, 1 unit of Taq DNA polymerase, and 100 ng of DNA template. QIAxcel Connect System capillary electrophoresis (QIAGEN, Hilden, Germany) with QIAxcel DNA High-Resolution Kit and QX Alignment Marker (15 bp/3 kb) were used for separating PCR products.

### 4.3. Statistical Analysis

The number of alleles (na), effective number of alleles (ne), Shannon’s information index (I), observed heterozygosity (Ho), expected heterozygosity (He), percentage of polymorphic loci (PPL), Nei’s genetic diversity index (Nei), principal coordinate analysis (PCoA), and the analysis of molecular variance (AMOVA) were evaluated using the GenAlEx 6.5 package [57]. In addition, the Mantel R test (pair-wise geographical and genetic distances correlation among the populations) was performed using GenAlEx software [57]. The amount of gene flow (Nm) between gene pools was calculated based on F_ST_ estimates, Nm = [(1/F_ST_) − 1]/4. The heterozygosity (H) and polymorphic information content (PIC) values were calculated using Gene-Calc software [58].

The unweighted pair-group method with arithmetic means (UPGMA) based on the genetic distance matrices was used for the reconstruction of the dendrogram of 15 populations in PAST software with 1000 bootstrap replications [59]. The STRUCTURE v2.3.4 program with the Bayesian clustering method was used to calculate the genetic structure of the 15 Central Asian *J.seravschanica* populations. The burn-in period and MCMC (Markov chain Monte Carlo) replications were set to 100,000, and the iteration number was set to 3. The results of the structure analysis were evaluated using the web-based program Structure Harvester [60], and the number of clusters (K) was obtained by employing Evanno et al. [61] and Jakobsson and Rosenberg’s [62] calculations in Structure Harvester.

## 5. Conclusions

In this study, 15 populations of *J. seravschanica* growing in the mountain ranges of four distinct Central Asian regions, including parts of Uzbekistan, South and North Kyrgyzstan, and Kazakhstan, were collected. The collected samples of 323 individuals from 15 natural populations were analyzed for genetic diversity and population structure using 11 polymorphic SSR markers. Genetic diversity results indicate that *J. seravschanica* in this region has a 0.450 value, with the lowest recorded value in North Kyrgyzstan (0.430) and the highest in Uzbekistan (0.473). The AMOVA results suggest that 90.3% of this genetic variation exists within populations and 9.7% between populations. The STRUCTURE outputs revealed a poor clustering in this species, confirming our AMOVA results. The Nm gene flow index was 4.654, suggesting a relatively high rate of gene flow. Population 5, collected in South Kyrgyzstan, was closest to the Zeravshan River and located in the middle of Coordinate 1 and Coordinate 2 eigenvalues of the PCoA plot, which indicates this population’s importance to the origin of this species. Accordingly, the obtained results on genetic diversity in *J. seravschanica* populations elucidated the populations with a high level of genetic diversity.

## Figures and Tables

**Table 1:** Characteristics of 11 polymorphic microsatellite markers used in *Juniperus seravschanica*.

Locus	na	ne	I	Nei	H	PIC
Jce03	3	1.8	0.666	0.442	0.485	0.410
Jce04	4	1.7	0.625	0.442	0.495	0.393
Jce05	2	1.1	0.132	0.077	0.081	0.077
JT_04	3	2.0	0.779	0.511	0.564	0.499
JT_30	3	2.3	0.921	0.586	0.606	0.538
JT_33	2	1.7	0.578	0.417	0.440	0.343
JT_34	2	1.3	0.292	0.191	0.207	0.185
JT_37	5	2.9	1.170	0.688	0.705	0.653
JT_40	5	2.6	1.028	0.620	0.712	0.662
JT_46	3	2.0	0.729	0.466	0.615	0.537
JS54	3	2.0	0.813	0.508	0.508	0.456
Mean	3.2	1.9	0.703	0.450	0.493	0.432
SE	0.069	0.051	0.027	0.016	0.195	0.180

Notes: na—number of alleles per locus; ne—effective number of alleles; I—Shannon’s Information Index; Nei—Nei’s genetic diversity index; H—heterozygosity value; PIC–polymorphism information content; SE—Standard error.

**Table 2:** Assessment of the genetic diversity of studied *Juniperus seravschanica* populations.

Pop	Size	Origin	na	ne	Ho	He	Nei	PPL
Pop 1	11	Uzbekistan	2.6	2.0	0.711	0.593	0.474	91%
Pop 2	10		2.2	1.7	0.700	0.562	0.418	91%
Pop 3	11		2.4	1.9	0.802	0.605	0.433	82%
Pop 4	12		2.7	2.3	0.682	0.639	0.566	91%
Mean			2.5	2.0	0.724	0.600	0.473	89%
Pop 5	13	South Kyrgyzstan	2.3	1.8	0.664	0.535	0.411	82%
Pop 6	21		2.8	2.2	0.684	0.592	0.497	100%
Pop 7	21		2.5	1.8	0.680	0.562	0.414	91%
Pop 8	20		2.5	2.0	0.700	0.581	0.452	91%
Mean			2.5	1.9	0.682	0.567	0.443	91%
Pop 9	20	North Kyrgyzstan	2.6	1.8	0.705	0.556	0.431	91%
Pop 10	20		2.5	1.9	0.668	0.555	0.430	91%
Mean			2.5	1.9	0.686	0.555	0.430	91%
Pop 11	41	Kazakhstan	2.8	1.8	0.694	0.568	0.419	100%
Pop 12	40		3.0	2.2	0.675	0.579	0.482	100%
Pop 13	20		2.5	1.9	0.627	0.537	0.447	100%
Pop 14	20		2.5	1.9	0.718	0.561	0.407	82%
Pop 15	43		2.6	2.1	0.717	0.602	0.465	91%
Mean			2.7	2.0	0.686	0.569	0.443	94%
Total mean	323		2.6	1.9	0.695	0.575	0.450	91.5%
SE			0.069	0.051	0.019	0.012	0.016	1.65%

Notes: Pop—population; na—number of alleles per locus; ne—effective number of alleles; Ho—Observed Heterozygosity; He—Expected Heterozygosity; Nei—Nei’s genetic diversity index; PPL—the percentage of polymorphic loci; SE—Standard error.

**Table 3:** AMOVA results for 15 populations of *Juniperus seravschanica* based on 11 SSR markers.

Source	df	SS	MS	%	*p*	Est. Var.	F_ST_	Nm
Among populations	14	113.302	8.093	9.7%	<0.001	0.266		
Within populations	308	761.596	2.473	90.3%	<0.001	2.473		
Total	322	874.898		100%		2.738	0.050 *	4.654

df—degrees of freedom; SS—sum of squares; MS—mean squared; Est. var.—estimates of variance; %—percentage of variation; F_ST_—fixation index; Nm—gene flow (Nm) value. * *p* < 0.001; Nm = (1 − F_ST_)/4F_ST_.

**Table 4:** The population structure of *Juniperus seravschanica* from four regions of Central Asia using the K3 and K5 steps of the STRUCTURE package.

Population	Sample Size	Origin	K3 (%)			K5 (%)				
			Cluster 1	Cluster 2	Cluster 3	Cluster 1	Cluster 2	Cluster 3	Cluster 4	Cluster 5
Pop 1	11	UZ	27.3	9.1	63.6	18.2	45.5	9.1	18.2	9.1
Pop 2	10		20.0	0.0	80.0	10.0	10.0	10.0	60.0	10.0
Pop 3	11		18.2	9.1	72.7	0.0	0.0	9.1	72.7	18.2
Pop 4	12		50.0	0.0	50.0	0.0	8.3	41.7	0.0	50.0
Pop 5	13	SKG	53.8	0.0	46.2	30.8	7.7	0.0	30.8	30.8
Pop 6	21		85.7	4.8	9.5	4.8	4.8	19.0	9.5	61.9
Pop 7	21		61.9	9.5	28.6	4.8	52.4	19.0	14.3	9.5
Pop 8	20		30.0	0.0	70.0	15.0	15.0	10.0	50.0	10.0
Pop 9	20	NKG	25.0	10.0	65.0	15.0	15.0	0.0	60.0	10.0
Pop 10	20		15.0	15.0	70.0	20.0	15.0	5.0	55.0	5.0
Pop 11	41	KZ	19.5	12.2	68.3	24.4	29.3	4.9	39.0	2.4
Pop 12	40		45.0	7.5	47.5	7.5	25.0	15.0	25.0	27.5
Pop 13	20		90.0	0.0	10.0	5.0	35.0	5.0	0.0	55.0
Pop 14	20		30.0	0.0	70.0	25.0	10.0	5.0	25.0	35.0
Pop 15	43		72.1	9.3	18.6	2.3	16.3	16.3	11.6	53.5

Notes: UZ—Uzbekistan; SKG—South Kyrgyzstan; NKG—North Kyrgyzstan; KZ—Kazakhstan.

**Table 5:** Geographical coordinates of sampled *Juniperus seravschanica* populations in three Central Asian countries.

Country	Population	N (Latitude)	E (Longitude)	Altitude (m)	Sample Size	Geographic Location
Uzbekistan	Pop 1	38.272472	67.291861	2120	11	Southwestern Gissar range mountain of Baysuntau, Surkhandarya region
	Pop 2	38.563444	67.479750	2210	10	Basin of the Sangardak river, Surkhandarya region
	Pop 3	38.590361	67.970222	2200	11	Basin of the Shargun, Surkhandarya region
	Pop 4	39.593611	68.465222	2267	12	Basin of Kashkasu-Sai, Zaamin National Park, Turkestan Range
South Kyrgyzstan	Pop 5	39.966694	69.642000	1800	13	Sulukta, Leilek district
	Pop 6	39.758083	69.949278	2273	21	Baul, Leilek district
	Pop 7	40.055444	71.884389	2103	21	Tamasha gorge, Batken Region
	Pop 8	40.204806	72.352278	2293	20	Abshir-Sai gorge, Turkestan mountain Range
North Kyrgyzstan	Pop 9	41.499278	70.965833	2160	20	Jalal-Abad region, Chatkal gorge
	Pop 10	42.763917	71.822817	1420	20	Talas region, Kara-Archa gorge
Kazakhstan	Pop 11	42.168389	70.381722	1701	41	Turkestan region, Sairam-Ugam State National Nature Park (SNNP), Sairam gorge, right bank of the Sairamsu river
	Pop 12	42.155694	70.235750	1817	40	Turkestan region, Sairam-Ugam SNNP, Kaskasu gorge, left bank of the Sairamsu river
	Pop 13	42.179500	70.333556	1530	20	Turkestan region, Sairam-Ugam SNNP, Saryaygyr gorge
	Pop 14	42.331250	70.372583	1605	20	Turkestan region, Aksu-Zhabagly state nature reserve (SNR), Aksu canyon, right bank of the Aksu river
	Pop 15	42.416528	70.207417	2110	43	Turkestan region, Tyulkubas district, Aksu-Zhabagly SNR, Mashat gorge

**Table 6:** Characteristics of SSR primers used for the analysis of *Juniperus seravschanica*.

№	Primers	Motif	Primer Sequences (5′-3′)	Expected Size (bp)	Annealing T (°C)
1	Jce03	(AATAC)6	F: TGGTTTATTCATCAACGCCC R: TTCATCCAGTGTTAAGCATGTACC	101–121	61
2	Jce04	(AAGAG)7	F: TCTTTGCCTTGACTTCTCGG R: CAATAGCAGGAACACTAAACGG	100–110	61
3	Jce05	(ATATC)6	F: GCTGCTTTAGCTTCATGGACG R: CCAACTCTTGAACTCTAATCTGTACTGC	173–198	63
4	Jce08	(ATAC)10	F: TGGATTCTGAAATTTGTATGCAGC R: AAGCAATGACAAAGCAAAGGC	176–202	60
5	Jce09	(ATAC)8	F: TGTATATTTCTAGTCAAATGCCTTCC R: GATCTCCATTCATCTCTACAATCC	120–155	61
6	Jce12	(TA)6...(CATA)8	F: GCCAGATTCAAGGATAATTGG R: TTTAGCAACAGTACTATGCAAG	157–163	55
7	Jce13	(CATA)12	F: TGTTGTCATACCCCTGTGAGC R: GCAGTTGGATGAATTTTGTTG	200–230	55
8	JT_03	(CTT)8	F: ACCCTCTATAAGGATGCTACCATGA R: AAAAGATAGATTGATAAGTTGAAAGGG	140	55
9	JT_04	(AGA)7	F: CCAAGGAATGATCTAACCTTTGAA R: TGGGATGCATATCTTATCTTCCT	174–219	55
10	JT_30	(TCT)10	F: AATCCCCTATCCTTGCCAGT R: TCAACAATATCAGCAAGTAATGAGA	128–220	57
11	JT_33	(CT)11	F: GAGCTTCCTTTGTAGATTTTGGG R: GTAAGAAGACACCACTCAGTCGAT	177–285	57
12	JT_34	(AG)12	F: CATGCATGGGTTATAATAATAGAGATA R: TGGGCACAAATTTTAGTGTAATG	110	55
13	JT_37	(GT)14	F: GATGTTTGTATCATATCCTTGATTGG R: TCCACACCTATCGGGTTCAT	123	55
14	JT_38	(AC)14	F: CCAACAAGCCTCCACCCTAT R: CAAGTTTGGAAAGTGTGGTCA	114	55
15	JT_40	(CA)20	F: GGCCGCATGATCCATTACT R: TCGTAACGTAATGACATGTATAGTGC	98–150	54
16	JT_46	(AGG) 7	F: TGAGATCACCTACTTCCTAGTGGA R: CCACCAAGGGCATAGAGTTC	174–237	54
17	JS31	(ATG)5	F: TTGGCTAATGATGTGCTTGC R: ACCCAAGCTATGTGCAGGAT	330–354	60
18	JS54	(CAT)7	F: CTTGTGGTTAGTGGTTGGCA R: CACTCTCCCAGTGGTGGTTT	255–279	60

## Data Availability

All data are provided in the article.

## Supplementary Materials

The following supporting information can be downloaded at: https://www.mdpi.com/article/10.3390/plants12162961/s1, Table S1. Allelic status in eleven SSR markers for plants of fifteen populations of *Juniperus seravschanica* (bp). Table S2. The population structure for samples in *Juniperus seravschanica* collected in Kazakhstan, Kyrgyzstan, and Uzbekistan at K2, K3, and K5 steps based on STRUCTURE analysis. Figure S1. The UPGMA dendrogram of fifteen *Juniperus seravschanica* populations using eleven SSR markers and Nei’s genetic distance values. Figure S2. The optimal K steps for fifteen populations of *Juniperus seravschanica* using 11 SSR markers and the STRUCTURE Harvester package.
