# Supplementary figures and images for: Genetic Diversity and Population Structure of Juniperus seravschanica Kom. Collected in Central Asia
Source: Plants (Basel). 2023 Aug 16;12(16):2961. doi: 10.3390/plants12162961 (PMC10459705; doi:10.3390/plants12162961)

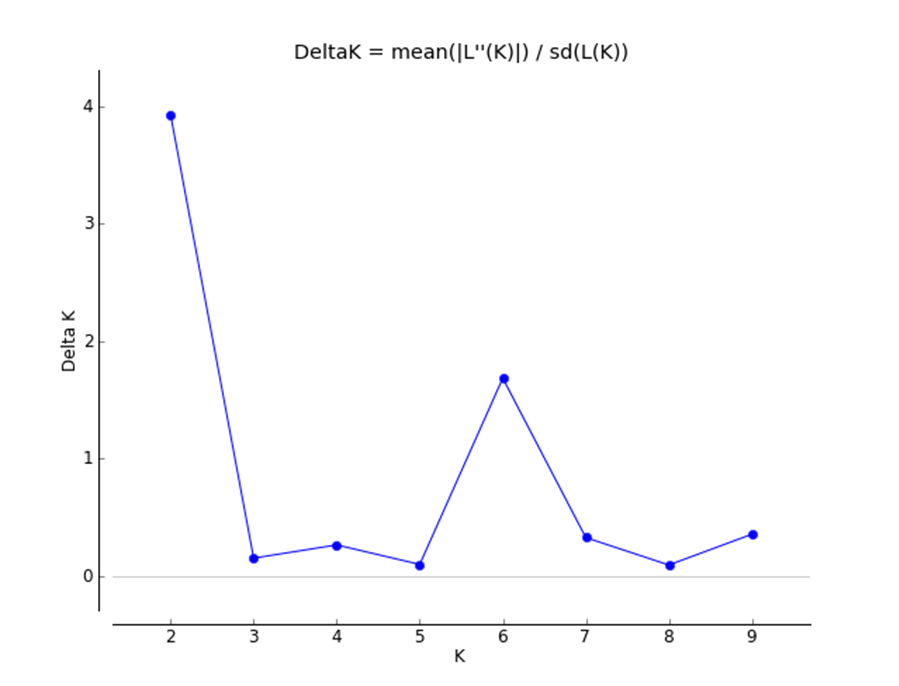

Supplement: Supplementary file 1 [file plants-12-02961-s001.zip › Figure S2.png]

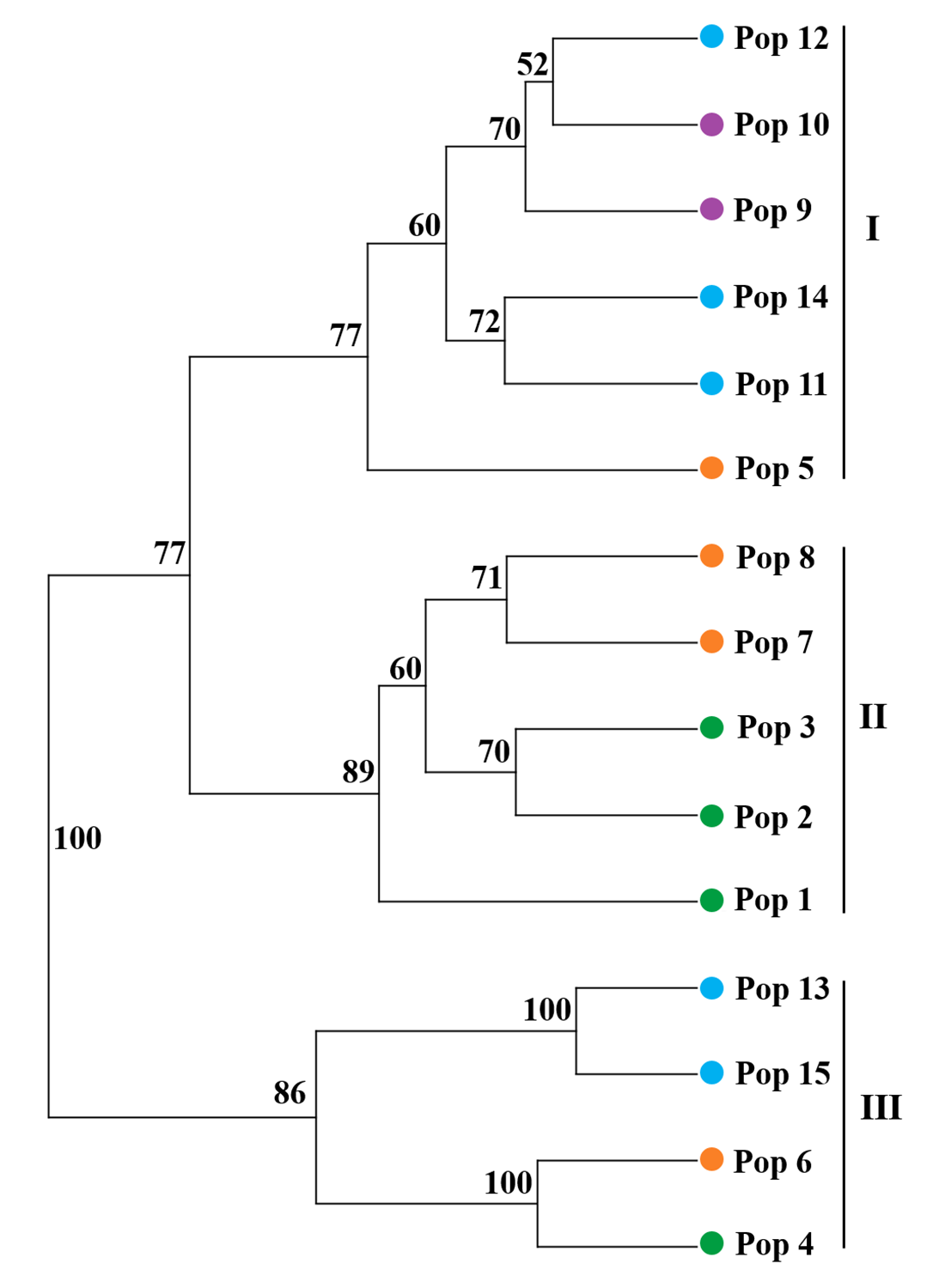

Supplement: Supplementary file 1 [file plants-12-02961-s001.zip › Figure S1.png]
